# The bovine chemokine receptors and their mRNA abundance in mononuclear phagocytes

**DOI:** 10.1186/1471-2164-11-439

**Published:** 2010-07-19

**Authors:** Stephanie Widdison, Nazneen Siddiqui, Victoria Easton, Freya Lawrence, George Ashley, Dirk Werling, Michael Watson, Tracey J Coffey

**Affiliations:** 1 Institute for Animal Health, Compton, Newbury, Berkshire, RG20 7NN, UK; 2 The Royal Veterinary College, Hawkshead Lane, North Mymms, Hatfield, Hertfordshire, AL9 7TA, UK

## Abstract

**Background:**

The chemokine and chemokine receptor families play critical roles in both the healthy and diseased organism mediating the migration of cells. The chemokine system is complex in that multiple chemokines can bind to one chemokine receptor and *vice versa*. Although chemokine receptors have been well characterised in humans, the chemokine receptor repertoire of cattle is not well characterised and many sequences are yet to be experimentally validated.

**Results:**

We have identified and sequenced bovine homologs to all identified functional human chemokine receptors. The bovine chemokine receptors show high levels of similarity to their human counterparts and similar genome arrangements. We have also characterised an additional bovine chemokine receptor, not present in the available genome sequence of humans or the more closely related pigs or horses. This receptor shows the highest level of similarity to CCR1 but shows significant differences in regions of the protein that are likely to be involved in ligand binding and signalling. We have also examined the mRNA abundance levels of all identified bovine chemokine receptors in mononuclear phagocytic cells. Considerable differences were observed in the mRNA abundance levels of the receptors, and interestingly the identified novel chemokine receptor showed differing levels of mRNA abundance to its closest homolog CCR1. The chemokine receptor repertoire was shown to differ between monocytes, macrophages and dendritic cells. This may reflect the differing roles of these cells in the immune response and may have functional consequences for the trafficking of these cells *in vivo*.

**Conclusions:**

In summary, we have provided the first characterisation of the complete bovine chemokine receptor gene repertoire including a gene that is potentially unique to cattle. Further study of this receptor and its ligands may reveal a specific role of this receptor in cattle. The availability of the bovine chemokine receptor sequences will allow further characterisation of the function of these genes and will confer wide-reaching benefits to the study of this important aspect of the bovine immune response.

## Background

The chemokine system has been shown to play a crucial role in both homeostasis, for example in lymphoid organogenesis and leukocyte maturation [1,2], and disease mechanisms. The system is complex and relies on the chemokine ligand binding to its chemokine receptor, with additional complexity arising from the fact that multiple chemokines can bind a single receptor and *vice versa*. The distinction between roles in homoeostasis and disease has been used as a means of functionally classifying both chemokines and chemokine receptors, although several chemokines have both homeostatic and inflammatory functions [3]. Inflammatory chemokines and their receptors have been demonstrated to have a role in the immune response to a myriad of pathogens both in humans and in other species. Homeostatic chemokines are generally constitutively expressed whereas the inflammatory chemokines are up-regulated following stimulation of the cell, for instance by cytokines or pathogens. It has recently been demonstrated that many of the inflammatory chemokines and their receptors exist in clustered groups in the mammalian genome and it is thought that these clustered chemokines have evolved relatively recently in evolutionary terms [4,5]. These inflammatory, clustered chemokines also tend to share functional properties, for example the CXCL chemokines, previously named Gro chemokines, are all capable of attracting neutrophils. This provides the chemokine system with an inherent robustness whereby the impairment of function in one chemokine can be overcome through the deployment of a second chemokine with similar properties, a capability aided by the inherent promiscuity of the chemokine system.

Both the chemokines and their receptors are grouped into four families, CC, CXC, XC and CX_3_C chemokines, depending on the location of C terminal cysteine residues in the chemokines with the receptors classified based on the chemokine family they bind. The chemokine receptors are G protein-coupled receptors with a conserved seven hydrophobic transmembrane structure and an extracellular N-terminus and intracellular C-terminus. The C-terminus is known to be involved in signalling following binding of the ligand, however there seems to be no consistent ligand binding mechanism. The chemokines studied to date use various combinations of the N-terminus and different extracellular loops of the transmembrane complex in order to bind the receptor [6].

In humans the chemokine receptor system has been well characterised. There are currently 18 known human chemokine receptors; CCR1-10, CXCR1-6, XCR1 and CX_3_CR1 [7] as well as other related receptors which bind chemokines but act as decoy receptors [8]. The availability of the bovine genome [9,10] has made it possible to begin to unravel the bovine chemokine and receptor system, however there are still areas of the genome which are yet to be properly assembled and at present many of the bovine homologs to the chemokine receptors are based on predictions from the genome sequence with no supporting experimental evidence. Experimentally confirmed sequences for bovine chemokine receptors are currently available in the public databases for *CCR1 *(NM_001077839), *CCR4 *(NM_001100293), *CCR5 *(NM_001011672), *CCR7 *(NM_001024930), *CCR9 *(NM_001098068), *CXCR1 *(EF597244), *CXCR2 *(NM_001101285), *CXCR3 *(NM_001011673), *CXCR4 *(NM_174301), *CXCR5 *(NM_001011675) and *CXCR6 *(NM_001014859). Cattle may also have at least one chemokine receptor not present in the human genome; C-C chemokine receptor type 1-like (NM_001075921).

In the case of the inflammatory chemokine receptors, differences between cattle and human receptor repertoires may have significant effects on the immune response. Following sequencing of the bovine genome, several key differences between the human and bovine immunome became apparent [9,10]. Firstly a newly discovered set of interferon (IFN) genes were identified which have been provisionally called the IFNX genes. Cattle also seem to have more IFN genes generally than other species, for example cattle have multiple copies of *IFN*β and *IFN*Ω [11]. Ruminants are also known to possess a unique IFNT family which are thought to have a role in pregnancy [12]. These differences between ruminants and other species may be due to major physiological differences in tissues such as the gut. The presence of increased numbers of microorganisms within the gut of ruminants renders them more susceptible to opportunistic infection. Cattle and other ruminants may therefore have evolved a more complex immune system to deal with the increased threat both from a physiological standpoint and due to the herd structure of these animals' populations which increase the risk of animal to animal transmission.

We present here a comprehensive investigation of the bovine chemokine receptor repertoire and their mRNA abundance by mononuclear phagocytes; macrophages (Mφ), dendritic cells (DC) and their precursors the monocytes (Mo). These cells are crucial players in the immune response to various pathogens and the chemokine receptors expressed by these cells will determine their chemotactic migration within an animal. We also report analysis of a chemokine receptor, CCR1L, which may be unique to cattle.

## Methods

### Identification of chemokine receptor genes in the bovine genome

The current bovine genome assembly Btau_4.0 available at the ENSEMBL genome browser http://www.ensembl.org was screened for bovine homologs to the 18 known human chemokine receptors using BLAST. We also screened available bovine sequences in the NCBI database http://www.ncbi.nlm.nih.gov for homologous sequences. The ENSEMBL browser was used as the basis for the examination of synteny and for gene structure prediction.

### Confirmation of bovine chemokine receptor cDNA sequences

As many of the currently identified bovine chemokine receptor sequences are predicted from the genome we set out to confirm their full length coding sequences (CDS). All receptors were amplified from cDNA produced from RNA extracted from Holstein Friesian cattle; CCR1, CCR1L, CCR2, CCR3, CCR4, CCR9, CCR10, CXCR3, CXCR4 and CXCR6 were isolated from lysed whole blood cells, CCR5 from blood-derived DC, CCR6 and CXCR5 from blood-derived B-cells, CCR7 and CX_3_CR1 from blood-derived Mo, CCR8 from a spleen tissue sample, CXCR1 and CXCR2 from a blood-derived granulocyte/Mo preparation and CXCR6 and XCR1 from a lymph node tissue sample. Blood-derived cells; lysed whole blood cells, Mo, DC and Mo/granulocytes were isolated as described previously [13,14], with the exception that DC were cultured for seven days with a media change after three days. For isolation of B-cells, PBMC were prepared from blood [14], then B-cells were isolated using the monoclonal antibody CC51, recognising the surface antigen CD21 [15], and anti-mouse IgG2a+b MicroBeads (Milltenyi-Biotech, Camberley, UK). RNA was isolated by lysing cells with 4 M guanidine thiocyanate followed by RNA extraction using the RNeasy mini kit (Qiagen, Hilden, Germany). Tissue samples were processed for RNA extraction as described previously [16]. The quality and quantity of the RNA were assessed using the Ultrospec 2100 pro spectrophotometer (GE Healthcare Ltd., Chalfont St Giles, UK). cDNA was prepared using Superscript II, Superscript III (Invitrogen Ltd., Refrenshew, UK) or RevertAid H Minus (Fermentas, York, UK) reverse transcriptases.

PCR amplification of full-length CDS of chemokine receptors was carried out using GoTaq DNA polymerase (Promega Co., Southampton, UK), Taq DNA polymerase (Invitrogen Ltd.) or the Expand High Fidelity^PLUS ^PCR System (Roche Diagnostics GmbH, Mannheim, Germany). Primers used for amplification are shown in Additional file 1; Supplemental Table S1. PCR products were then cloned into TA cloning vectors; pCR2.1-TOPO (Invitrogen Ltd.), pGEM-T Easy (Promega Co.) or pDrive (Qiagen). Sequencing of inserts was performed using the using BigDye1 Terminator v3.1 Cycle Sequencing Kit (Applied Biosystems, Warrington, UK) on an ABI PRISM 310 Genetic Analyser (Applied Biosystems). Primers used for sequencing are shown in Additional file 1; Supplemental Table S1. Sequence analysis was performed using the SequencherTM package version 4.1.4 (Gene Codes Corporation, Ann Arbor, USA).

### Phylogenetic analysis of identified bovine chemokine receptors

The amino acid sequences of identified bovine chemokine receptors and available full length homologous sequences from humans, sheep, pigs and horse were aligned using ClustalW [17], and a phylogenetic tree constructed using PHYLIP [18]. Evolutionary history was inferred using the Maximum Parsimony (MP) method, the percentage of replicate trees in which the associated taxa clustered together in the bootstrap test (500 replicates) are shown next to the branches.

### Real-time PCR evaluation of bovine chemokine receptor mRNA abundance in mononuclear phagocytes

Quantitative real-time PCR on triplicate samples was carried out using TaqMan FAST Universal PCR Mastermix (Applied Biosystems, Warrington, UK) on an ABI Prism 7500 Fast Real-Time PCR System (Applied Biosystems) using 100 ng cDNA as the starting template. Primers and probes used for real-time PCR analysis of CCR1, CCR3, CCR5, CXCR1, CXCR2 and GAPDH have previously been reported [19,20]. Primers and probes for the other bovine chemokine receptors are given in Additional file 1; Supplemental Table S2. Amplification consisted of an initial denaturation step of 95°C for 20 seconds, followed by 40 cycles of 95°C for 3 seconds and 60°C for 30 seconds. The transcript copy number for a given gene was calculated by comparison with plasmid standard curves containing known copy numbers of target genes. Relative mRNA abundance values were then calculated according to the 'Relative Quantitation of Gene Expression Experimental Design and Analysis: Relative Standard Curve Method' (Applied Biosystems Technical Bulletin: 'Guide to Performing Relative Quantitation of Gene Expression Using Real-Time Quantitative PCR'). For each target gene, mRNA abundance levels were normalised to the housekeeping gene GAPDH according to the formula (Copy No. Target/Copy No. GAPDH).

### Statistical analysis of quantitative real-time PCR data

Analysis of data was carried out using Microsoft^® ^Excel 2007 (Microsoft Co., Redmond, WA, USA) and GraphPad Prism 5.01 for Windows (GraphPad Software, San Diego California USA, http://www.graphpad.com). Differences between groups were determined by repeated measures one-way analysis of variance (ANOVA) followed by Tukey's multiple comparison tests.

## Results

### Characterisation of bovine homologs to human chemokine receptors

In the human system, ten CCR receptors (CCR1-10), six CXCR receptors (CXCR1-6) and the unique XCR1 and CX_3_CR1 receptors have been characterised. We were able to identify bovine homologs to all of these receptors by bioinformatic analyses of available bovine sequences and genome databases. We set out to confirm identified genes by amplification of transcripts from bovine mRNA. For the CCR- chemokine receptors, sequences for *CCR1*, *CCR4*, *CCR5*, *CCR7 *and *CCR9 *have all been published however the other CCR-receptor sequences are only predicted from the genome. For the published CCR-chemokine receptor sequences we were able to confirm the sequence data available, although some synonymous differences were detected, likely due to breed-specific differences. We did however, detect six amino acid differences in *CCR9 *compared to the published sequence (NP_001091537) (Additional file 1; Supplemental Figure S1). These were located in the transmembrane regions and the extracellular carboxy- and intracellular amino-termini of the protein and may also reflect breed-specific differences as the available sequence data comes from a Hereford animal whilst our data is from a Holstein Friesian animal. Whether these amino acid changes would confer functional differences in the CCR9 receptor remains to be determined. Our sequence for *CCR9 *has been submitted to GenBank (GU936964).

We also confirmed coding sequences for the CCR-chemokine receptors that are currently only available as predicted sequences; *CCR2*, *CCR3*, *CCR6*, *CCR8 *and *CCR10*. These have also been submitted to GenBank (*CCR2 *- GU936960, *CCR3 *- GU936961, *CCR6 *-GU936962, *CCR8 *- GU936963, *CCR10 *- GU936965). We detected synonymous nucleotide changes in *CCR2 *and *CCR10 *compared to the published sequences. *CCR3 *has two transcript variants in the NCBI database (XM_869148, XM_589282) and one truncated transcript in the ENSEMBL database (ENSBTAT00000001762). The *CCR3 *sequence we amplified (GU936961) is identical to transcript variant 1. *CCR6 *has one transcript variant in the NCBI database (XM_597941) and three in the ENSEMBL database (ENSBTAT00000037034, ENSBTAT00000056219, ENSBTAT00000053991). The sequence we identified (GU936962) was very similar to the NCBI transcript except for one amino acid change located in the second extracellular loop. Our *CCR10 *sequence (GU936965) is identical at the amino acid level to the predicted sequence (XP_584874) however minor differences exist at the nucleotide level.

Sequences for *CXCR1-5 *have previously been published. The sequences we detected mostly had synonymous differences compared to those published except for *CXCR5 *(GU936966) which had three amino acid changes compared to the published sequence (NP_001011675); one in the extracellular amino-terminus, one in the second extracellular loop and one in the intracellular carboxy-terminus. There are multiple sequences available for CXCR6 in the NCBI database (AAX08649, NP_001014859, AAX08913), and one in the ENSEMBL database (ENSBTAG00000015708) which is identical at the amino acid level to NP_001014859 although synonymous nucleotide differences are present between the two sequences. All three sequences deposited in the NCBI database differ at the amino acid level and our sequence (GU936967) is also different from these. The sequences deposited in GenBank are all from pooled samples which may account for the sequence differences seen. The sequence for *XCR1 *is currently only predicted from the database (XM_870210). We amplified an *XCR1 *transcript (GU936968) which has one amino acid change, compared to the predicted sequence, in the second intracellular loop. Experimentally confirmed sequence has been published for CX_3_CR1 (AAI48891) but this is from a Hereford animal. Our sequence (GU936969) differed from the published sequence by three amino acids, located in the extracellular amino-terminus, the second transmembrane domain and the fourth transmembrane domain. These are likely to be breed-specific differences.

**Figure 1:**
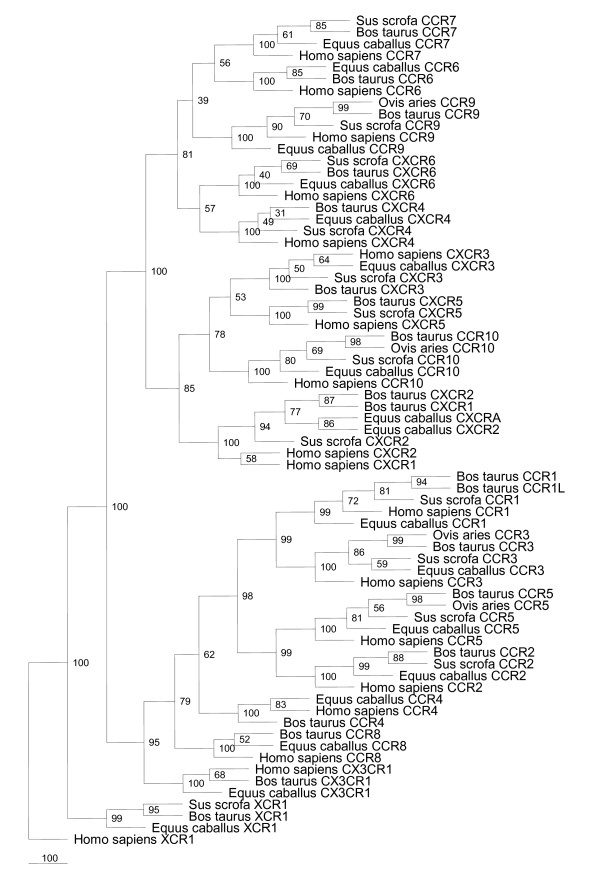
**Phylogenetic analysis of full-length chemokine receptor sequences from human, bovine and other related species**. Where differences exist to the published sequences, bovine sequences are those obtained by us. No equine CXCR1 has been identified, however a homologous sequence to CXCR1 has been identified (XP_001491062) and here has been designated CXCRA. See Methods for tree construction methods.

When compared to chemokine receptor sequences from humans and more related species (Figure 1) the bovine receptors generally group well with their homologs indicating a common evolutionary history for most of the chemokine receptors. A significant exception comes in the form of the two CXCL8 receptors, CXCR1 and CXCR2. Phylogenetic analysis reveals that for the bovine and equine CXCR1 and CXCR2 sequences, the two sequences are more closely related at the intraspecies level than to their designated homologs in other species (Figure 1). As has been reported previously, this is due to bovine CXCR1 and CXCR2 having identical carboxy-termini, whereas in humans this intracellular domain differs between the two receptors [21].

### Characterisation of CCR1L, a chemokine receptor potentially unique to cattle

**Figure 2:**
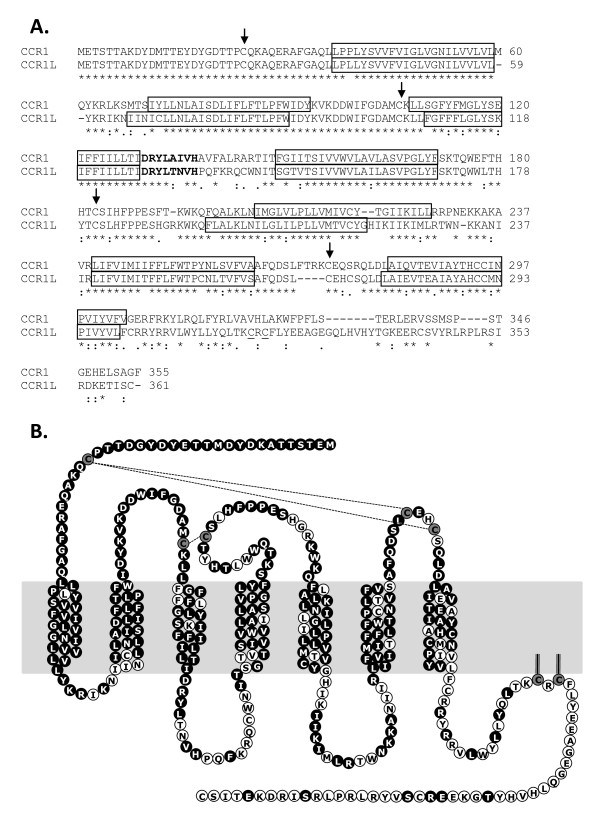
**Homology between bovine CCR1 and CCR1L**. A) Amino acid alignment of bovine CCR1 and CCR1L. Conserved extracellular cysteine residues are arrowed and the DRY motif is in bold. Boxed areas indicate transmembrane domains as predicted by SMART analysis [22,23]. B) Predicted membrane topology of CCR1L. Black circles indicate homology to CCR1, white circles indicate differences between CCR1 and CCR1L, grey circles indicate extracellular and potentially palmitoylated cysteine residues. Dotted lines indicate potential disulphide bridges.

During our search for bovine homologs of human chemokine receptors we identified an additional chemokine receptor that was not present in humans. This receptor was previously designated C-C chemokine receptor type 1-like (NM_001075921), henceforth abbreviated to CCR1L. *CCR1L *is located adjacent to *CCR1 *in the bovine genome and is most closely related to *CCR1 *when compared to all bovine chemokine receptors (Figure 1, 2), potentially representing a gene-duplication event in cattle. SMART analysis [22,23] revealed the existence of seven transmembrane domains, common to all chemokine receptors. We also identified the characteristic DRY motif, located in the second intracellular loop, which is required for activation [24,25]. By predicting the membrane topology of CCR1L and mapping the amino acid differences onto this topology (Figure 2B) it becomes apparent that the extracellular amino-termini of CCR1L and CCR1 are identical, however the rest of the protein, particularly the intracellular loops and the intracellular carboxy-termini are markedly different. There are four cysteine residues present in CCR1 which have important roles in maintaining conformation, ligand binding, signalling and trafficking of chemokine receptors to the cell membrane [26,27]. In addition to these conserved cysteines between CCR1 and CCR1L in cattle, CCR1L has an additional cysteine in the third extracellular loop and an additional pair of cysteines in the intracellular tail (Figure 2B). Homologs to *CCR1L *are not present in the available ENSEMBL pig or horse genome assemblies and preliminary attempts to detect *CCR1L *in water buffalo, sheep and goats were unsuccessful (data not shown). It is therefore possible that CCR1L represents a chemokine receptor unique to cattle, however further investigations are required to confirm this.

### Chemokine receptor expression by bovine mononuclear phagocytic cells

**Figure 3:**
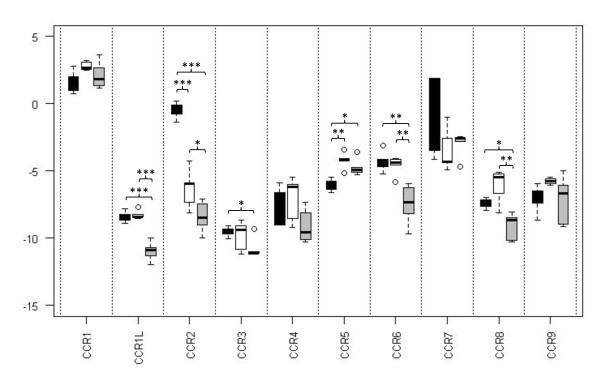
**Real-time PCR analysis of CC-chemokine receptor mRNA abundance by bovine blood-derived Mo, Mφ and DC**. Boxplots of average Log(2) normalised value (see Methods). Black bars - Mo, white bars - Mφ, grey bars - DC. * - P-value < 0.05, ** - P-value < 0.01, ***-P-value < 0.001.

**Figure 4:**
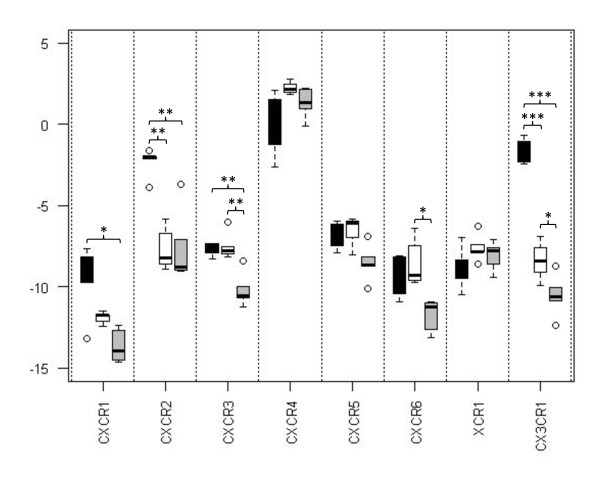
**Real-time PCR analysis of CXC-, XC- and CX**_**3**_**C-chemokine receptor mRNA abundance by bovine blood-derived Mo, Mφ and DC**. Boxplots of average Log(2) normalised value (see Methods). Black bars - Mo, white bars - Mφ, grey bars - DC. * - P-value < 0.05, ** - P-value < 0.01, ***-P-value < 0.001.

As little is known about the expression levels of bovine chemokine receptors we set out to determine mRNA abundance in a group of cells important in the immune response, the mononuclear phagocytes. These cells, which include the Mφ, DC and their precursor cell the Mo, are thought to express chemokine receptors during both homeostasis and disease as a means of targeting to sites of infection. Real-time PCR assays were developed in order to measure the mRNA abundance of all identified bovine chemokine receptors in the three cell types isolated from five animals. mRNA abundance levels of the CCR-receptors were quite variable with some receptors, such as *CCR1*, being expressed at high levels when compared to others such as *CCR3 *and *CCR4 *(Figure 3). No *CCR10 *mRNA was detected for any of the mononuclear phagocytes, however mRNA was detected for other bovine immune cells (data not shown). Some CCR receptors, *CCR1*, *CCR4*, *CCR7 *and *CCR9*, were not expressed differently between the cell types, whilst others showed cell-specific levels of mRNA abundance. mRNA abundance of the CXCR-, XCR- and CX_3_CR-receptors also showed considerable variability with *CXCR1*, *CXCR2*, *CXCR3*, *CXCR6 *and *CX*_*3*_*CR1 *showing variability between the three cell types (Figure 4). Considering the receptors as a whole, differentiation of Mo to Mφ resulted in an increase in mRNA abundance for *CCR5 *and a decrease in *CCR2*, *CXCR2 *and *CX*_*3*_*CR1*. DC also showed higher levels of *CCR5 *mRNA abundance than Mo and lower levels of *CCR1L*, *CCR2*, *CCR3*, *CCR6*, *CCR8*, *CXCR1*, *CXCR2*, *CXCR3 *and *CX*_*3*_*CR1*. Comparing Mφ and DC, Mφ demonstrated higher abundance of *CCR1L*, *CCR2*, *CCR6*, *CCR8*, *CXCR3*, *CXCR6 *and *CX*_*3*_*CR1*.

## Discussion

The chemokine receptors are thought to have originated from a single ancestral gene with individual genes arising due to subsequent gene duplication events [28]. Many of the chemokine receptors are clustered across the genome and these proximal chemokine receptors also tend to be closely related. Generally the chemokine receptor family is well conserved between mammalian species, as can be seen by a comparison of the well-studied human and murine chemokine receptor genes, however some differences do exist indicating ongoing evolution of the chemokine receptor system. Here for the first time, we have characterised the full complement of bovine homologs to known human chemokine receptors and in doing so have also further characterised an additional chemokine receptor not found in humans. Generally the bovine chemokine receptors showed high homology to their human counterparts and chromosomal organisation was well conserved. However, differences were identified when comparing the bovine and human chemokine receptors. As reported previously, the bovine CXCR1 and CXCR2 display a higher degree of homology to each other than the human receptors, due to the lack in cattle of a differing carboxy-terminal in cattle as is found in humans [21]. Despite this, the two bovine receptors seem to have similar functional properties to the human receptors. Without further study the functional relatedness of the complete complement of human and bovine chemokine receptors cannot be assumed however the high degree of sequence similarity between the receptors suggests many properties may be shared between species. As expected, there was also a high degree of similarity between the chemokine receptors of cattle and those from other ruminants and members of the Cetartiodactyla, although limited sequence data are available for these species. There is a significant difference between the *XCR1 *gene of pigs, cattle and humans in that much of the 5' un-translated region of porcine *XCR1 *is shared with the first exon of *CCR1 *[29]. In addition, horses have an extra CCR-chemokine receptor that seems to have formed as a result of recombination between *CCR2 *and *CCR5 *[30].

One major difference between the bovine and human chemokine receptor repertoire is in the presence of an additional CCR-chemokine receptor, C-C chemokine receptor type 1-like, here called CCR1L. This receptor is located adjacent to *CCR1 *in the bovine genome and may have arisen as a result of a gene duplication event as indicated by phylogenetic analysis. *CCR1L *is not present in the genome sequences of humans, horses or pigs and so this may represent a relatively recent evolutionary event, however whether CCR1L is unique to cattle is at present unknown. Bovine CCR1 and CCR1L share identical amino-termini however the remainder of the sequence differs and overall the two sequences share only 78% nucleotide identity , 66% amino acid identity and 73% similarity. Whether the CCR1L receptor is functional is not yet clear, however the gene is transcribed and analysis of transcriptional levels in mononuclear phagocytes revealed that *CCR1L *is also transcribed differently to *CCR1 *in these cells. CCR1L also possesses a number of features characteristic of functional chemokine receptors including the DRY motif, which is lacking in the non-signalling receptors D6 and DARC [31], and the NPXXY motif in transmembrane region 7 which is thought to be important for G protein-coupled receptor activation [32]. We also identified cysteines in the extracellular loops of CCR1L which are critical for normal chemokine receptor function [26,27]. Unusually, CCR1L has two cysteine residues in the third extracellular loop. The additional cysteine in the third extracellular loop may bind to the extracellular amino-terminus as cysteines in this region are known to form disulphide bridges with the amino-terminus [27]. However without further study the functional significance of these two cysteine residues in the third extra-cellular loop is unknown. Although not present in CCR1, most other chemokine receptors also have cysteine residues in their carboxy-termini. These cysteines, normally located 12-25 amino acids from the transmembrane region as found for CCR1L, are suggested to be palmitoylated, particularly when surrounded by clusters of amino acids that are hydrophobic and positively charged as for CCR1L [33]. These cysteines effectively anchor the intracellular carboxy-terminus to the plasma membrane and hence create a fourth intracellular loop (Figure 2). For CCR5 it is has been shown that palmitoylation is essential for transport to the plasma membrane and for downstream signalling activation and may also be required for phosphorylation of the receptor [34-36].

At present the ligands, if any, for CCR1L are unknown however we can begin to speculate about possible ligands based on sequence similarity at the important binding regions of the chemokine receptors. The amino termini of CCR1 and CCR1L in cattle are identical and it has been shown by chimera studies that this domain in human CCR1 confers a degree of ligand specificity [37] thus it could be speculated that CCR1 and CCR1L bind the same ligands. However, full activation of CCR1 seems to require additional sites, in particular CCL3 has been shown to interact with CCR1 at the second extracellular domain. CCR1L and CCR1 differ considerably in this region suggesting that there may be differences in ligand binding between the two receptors. Without further study it is impossible to know exactly which ligands bind CCR1L, however given the restriction of CCR1L to cattle and possibly other related species it is interesting to note that cattle have a unique CC-chemokine, regakine, which seems to synergise with CXCL8 to increase neutrophil migration but for which no receptor has currently been identified [38,39]. It is therefore possible that CCR1L and regakine represent a chemokine:receptor pairing that is absent from humans but present in cattle and potentially other ruminants. Given the importance of chemokines in the immune response, the restriction of CCR1L and regakine to cattle may relate to the differing immune requirements of humans and cattle.

There are several papers reporting the chemokine receptor repertoire of mononuclear phagocytic cells and the changes in their expression during maturation from Mo to antigen-presenting cells in humans. Mo have been shown varyingly to express CCR1, CCR2, CCR5, CCR7, CCR8, CXCR1, CXCR2 and CX_3_CR1 [40-48] which aid in their dissemination from the bone marrow into the tissues. Upon maturation to Mφ, Mo down-regulate CCR2 and increase expression of CCR1 and CCR5 [46,49]. DC have been shown to express a range of chemokine receptors however the expression level depends on the maturation state of the cell. Immature DC express a range of receptors for the inflammatory chemokines including CCR1, CCR2, CCR5, CCR6 and CXCR1 which guide the cell to sites of inflammation however maturation results in a down-regulation of many of these receptors and an up-regulation of receptors for constitutive chemokines such as CCR7 and CXCR4 [44,45]. This then allows the DC to traffic back to the lymph node in order to present antigen to T-cells. The down-regulation of CCR1 and CCR5 has been shown to be due to the release of chemokines by the DC, which CCR7 is resistant to [50]. Many of the receptors may only be functional under certain conditions, for example mononuclear phagocytes have been shown only to respond to CXCL8 following appropriate cytokine stimulation [51]. In the bovine system we were only able to assess expression levels at the mRNA level as no antibodies are currently available for bovine chemokine receptors. Studies in humans have revealed that for some chemokine receptors functional receptor levels are reflected at the mRNA level, for example the down-regulation of CCR2 and the up-regulation of CCR1 and CCR5 during the maturation of Mo to Mφ [46]. However for other chemokine receptors, such as the observed down-regulation in CCR1 and CCR5 by maturing DC, this was shown to be due to the redistribution of the receptor to an endocytic compartment rather than an actual change in total protein levels [50]. Despite our inability to measure protein levels, the mRNA abundance levels provide a useful insight into the chemokine receptor repertoire of bovine cells. At the mRNA level we were able to confirm the down-regulation of *CCR2 *and up-regulation of *CCR5 *seen in human cells upon maturation of Mo to Mφ, and in addition demonstrated the down-regulation in transcription of *CXCR2 *and *CX*_*3*_*CR1*. For DC we were only able to detect higher levels of *CCR5 *compared to Mo but demonstrated the down-regulation of a number of receptors including *CCR2*, *CCR6 *and *CXCR1 *which have been shown to be down-regulated upon maturation of human DC.

## Conclusions

In conclusion we present here for the first time a complete characterisation of the bovine chemokine receptor system. The bovine system shows many similarities to the well-characterised human and murine systems, with a couple of exceptions. Firstly, as reported previously, the CXCR1 and CXCR2 of cattle are much more closely related than those of humans and so may exhibit functional differences to the human receptors. Secondly, cattle possess the gene for an additional chemokine receptor not present in humans. This receptor shows some homology to CCR1 but possesses some amino acid differences that could result in the receptor differing in ligand binding or signalling to CCR1. We have also determined the mRNA abundance of all identified bovine chemokine receptors in mononuclear phagocytic cells and demonstrated similar trends in chemokine receptor repertoire to those in humans. The availability of chemokine receptor sequences will hopefully fuel the production of antibodies to these important immune modulators and allow more extensive characterisation of the bovine chemokine system. Further study of species-restricted chemokines and receptors may also reveal a facet of the chemokine system that is unique to cattle and other closely related species.

## Authors' contributions

SW participated in the design and coordination of the study, sequencing analysis, the design of real-time PCR assays and data analysis, carried out all of the expression analyses, and drafted the manuscript. NS participated in the design of real-time PCR assays. VE, FL and GA participated in the sequencing analyses. DW helped to draft the manuscript, MW participated in the data analysis. TJC participated in the design and coordination of the study and helped to draft the manuscript. All authors read and approved the final manuscript.

## Supplementary Material

Additional File 1 **Supplemental Figure S1 Amino acid alignment of sequenced CCR9 with published CCR9 sequence (NP_001091537)**. Amino acid alignment of bovine CCR9 sequenced as part of this study with the published bovine CCR9 sequence illustrating amino acid differences. **Supplemental Table S1 **Primers used for PCR and sequencing. Table of primers used in this study to PCR and sequence bovine chemokine receptors. **Supplemental Table S2 **Primers and probes used for real-time PCR. Table of primers and probes used in this study to carry out real-time PCR analysis of bovine chemokine receptor expression. Click here for file
