# Supplementary material for: The bovine chemokine receptors and their mRNA abundance in mononuclear phagocytes
Source: BMC Genomics. 2010 Jul 19;11:439. doi: 10.1186/1471-2164-11-439 (PMC3091636; doi:10.1186/1471-2164-11-439)
Supplement: Additional File 1 — Supplemental Figure S1 Amino acid alignment of sequenced CCR9 with published CCR9 sequence (NP_001091537). Amino acid alignment of bovine CCR9 sequenced as part of this study with the published bovine CCR9 sequence illustrating amino acid differences. Supplemental Table S1 Primers used for PCR and sequencing. Table of primers used in this study to PCR and sequence bovine chemokine receptors. Supplemental Table S2 Primers and probes used for real-time PCR. Table of primers and probes used in this study to carry out real-time PCR analysis of bovine chemokine receptor expression. [file 1471-2164-11-439-S1.DOC]

Supplemental Table S1. Primers used for PCR and sequencing

| **Gene** | **Forward Primer (5’-3’)** | **Reverse Primer (5’-3’)** |
| --- | --- | --- |
| *CCR1* | ATGGAAACTTCAACCACCGCAAAGG | TCAGAACCCAGCAGAGAGTTCATGC |
| *CCR1 INT* | GCTCAGAAGACCAAATGAGAAGAA | CCCAGAGAGCAACTTACACATG |
| *CCR1L* | GCCCACTGCCTCTAACAGC | CCATGTGGCTACAATTCCAGG |
| *CCR2* | GCATGGATGGCAATGATACATTCAG | CTATAAAGCAGCTGAGACTTCCTGTT |
| *CCR2 INT* | GAAGAAGCACAAGGCTGTGAG | GGATGATGAAGAAGATTCCACC |
| *CCR3* | ATGGCGACCTCAGCTGATG | CTAAAATACAGCAGAGAGTTCCTGC |
| *CCR3 INT* | TATTAAAACACTGCTGAGATGCCC | GTACAAGCCCATGTAATAGAGCC |
| *CCR4* | ATGAACCCCACGGATATAGCAGA | TTACAGAGCATCGCGGAGGT |
| *CCR4 INT* | AGATGATCTTTGCTGTGTGGTGGTC | AAGCCCAAACACCCACTGGTC |
| *CCR5* | ATGGATTATCAAACATCAACTCC | TCACAAGCCAACAGAGATTTC |
| *CCR5 INT* | ATGCTGGTTGTCCTCATCCT | AGATTTCCTGTTCTCCTGTGG |
| *CCR6* | ATGAATTCCACCAACATCTACGAT | TCACATGGTGAAGGAAGACGG |
| *CCR6 INT* | TGGAAGCTGCTGATGCTGG | CCTGCACGATGGCGATGTA |
| *CCR7* | ATGGACCTGGGGAAGCCAATGAA | CTACGGGGAGAAGGTGGTGG |
| *CCR7 INT* | CCTCGTCATCATCCGCACCTG | GGAAGCCTACCACCTCTGGG |
| *CCR8* | ATGGATTACACACCTGAGCCCAATT | TCACAGAATGTAGTCTACGGTGGAG |
| *CCR8 INT* | CCTATGCCACGCACGTCACAGAGA | CCGAATACCCACTGGTCCA |
| *CCR9* | ATGGTCCCCACAGAAGCCAC | TCAGAAGAAGAGAGCCCCCGA |
| *CCR9 INT* | CATCACACCCTGATCCAGGC | CAGGACCTGTAGAAGTTCATCTTATAC |
| *CCR10* | ATGGGGACTGAGCCCGC | CTAGTGGTCCCAGGAGGAGAAACT |
| *CCR10 INT* | CACGATGACTGAGACCAAGTG | GGCCTGCTATGCGCTCCTGGG |
| *CXCR1* | ATGACAATCATCCTGAAAGATTTATC | TCAGAGGGTAGTAGACGTGTTCCC |
| *CXCR1 INT* | CTATCAACCACCATACTCCGACC | GTATGGTGGTTGATAGGCCTCA |
| *CXCR2* | ATGGCTGAAACAAAATTTACTTCAA | TCAGAGGGTAGTAGACGTGTTCCC |
| *CXCR2 INT* | GGCCATCCACCCACCC | GCCAGGTTCAGCAGGTAGAC |
| *CXCR3* | ATGGTCCCTGAGATGAGTGAA | TCACAGGCCTGAGTAGGAGG |
| *CXCR3 INT* | TGAGATGAGTGAACGCCAAG | CCCACAAAGGCATAGAGCA |
| *CXCR4* | GCATGGAAGGGATCCGTATATTCACTT | TTAGCTGGAGTGAAAACTTGAAGATTCAG |
| *CXCR4 INT* | TTATCATCTCCAAGCTGTCCCACT | AAGCGTGAGGACAAACAGGAGGA |
| *CXCR5* | ATGAACTACCCTCTCACTCTGGACAT | CTAGAACGTGGTGAGAGAGGTGG |
| *CXCR5 INT* | ATGACTCCCTGCCGCACT | ATGGAGAGGAGGCGGC |
| *CXCR6* | ATGGCTGAGTACAACTACGAAGACC | CTACAGGTGGAACATGCTGGT |
| *CXCR6 INT* | CGGAAGCACAAGTCTCTGAA | AACCACCCAGATGGACGACG |
| *XCR1* | ATGGAGCCCTCAGACATCCC | TCAATAGAAGGAGATGCCCTCGTAGG |
| *XCR1 INT* | GGTCATCCTGTTCTGCTACGTG | CCGACAGGTAGCGATGGATG |
| *CX_3_CR1* | ATGCACACCACCCTCCCTG | TCAGAGAAGGATGGACGCATCTCC |
| *CX_3_CR1 INT* | ACTTCAGAATCATGCAGACATTGT | CACGGTCCTGTTGCTCATGGAGT |

INT – Internal primer used for sequencing only

Supplemental Table S2. Primers and probes used for real-time PCR

| **Gene** | **Forward Primer (5’-3’)** | **Reverse Primer (5’-3’)** | **Probe 5’-3’** |
| --- | --- | --- | --- |
| *CCR1L* | GTACCGCAGGGTTCTATGGT | CGTTCTTCCTTGCCTGTGTA | TGACCCTCTCCTGCCTCCTCG |
| *CCR2* | CACAATGTGCTTCCCACATC | GAGCTGTGCTTCGATTTGTC | CCCTGCCGAAAGACCAGCGT |
| *CCR4* | GCCTCTGCAAGCTGATTTCT | AGTCAAGGTCCTGGCTCTCA | TACCTGGCCATCGTGCACGC |
| *CCR6* | CTGCTCTTCGGCTTCTTCAT | AGTTCTGCGCCTGGACTAAG | CCGCTGGTGTTCATGATCTTCTGC |
| *CCR7* | AGCCAATGAAGAACGTGTTG | AATCGTCCGTGACCTCATCT | TTTCCAGGTGTGCCTCTGCCA |
| *CCR8* | TTCTGGGTCCCTTTCAACAC | CTCATGACACACCCATCCAG | TTCCCTGCATGACATGCACGTC |
| *CCR9* | CACAGAAGCCACAAGCCTAA | TCTCACAGAAGTAGTCCGTGAAG | TGCCGTCGTACTCCATGGGC |
| *CCR10* | CCGCAGAACAGGTCTCCT | GGGAGACACTGGGTTGGA | TCCAGGCCTTCAGTCGCGC |
| *CXCR3* | TGAGATGAGTGAACGCCAAG | GCACAAAGAGGAGGCTGTAGA | CCTGCCCACAGGACTTCAGCC |
| *CXCR4* | TTGCTGACATCAAGGAGGTG | ACCACGATGTGCTGAAACTG | TCCCAGCGACCTGTGGCTAGTG |
| *CXCR5* | ATGAACTACCCTCTCACTCTGGA | CTCCCTAATTCCTTGTACAGGTC | CATGGACCTCATGAACTACAACCTGGA |
| *CXCR6* | TTCCGGAAGCACAAGTCTCT | GATGAGCTTCACGAGGTTGA | CAGGAACACCGCCACCACCA |
| *XCR1* | TCCTGTACTGCCTGGTGTTC | GTCTGAGAGGCACAGGTTGA | TCAGCCTGGTGGGCAACAGC |
| *CX_3_CR1* | CCATCCTGACCACCTCAGTT | CATCTCCATCGCTGGTGTAA | TCCCGCCTGCTCCTTTGTGA |

Supplemental Figure S1

CCR9 MVPTEATSLIPNLSDDYSYDGTPPMEYDGNFTDYFCEKSHVRQFAGHFLPPLYWLVFIVG 60

NP_001091537 MVPTEATSLIPNLSDDYGYDGTLPMEYDGNFTDYFCEKSHVRQFAGHFLPPLYWLVFIVG 60

*****************.**** *************************************

CCR9 GVGNSLAILVYWYCTRVKTMTDMFLPNLAIADLLFLVTLPFWAIAAADQWKFQTFMCKVV 120

NP_001091537 GVGNSLVILVYWYCTRVKTMTDMFLLNLAIADLLFLATLPFWAIAAADQWKFQTFMCKVV 120

******.****************** **********.***********************

CCR9 NSMYKMNFYSCVLLIMCISVDRYIAIAQAMRAQMWRQKRLLYSKMVCFTIWVTAAALCLP 180

NP_001091537 NSMYKMNFYSCVLLIMCISVDRYIAIAQAMRAQMWRQKRLLYSKMVCFTIWVTAAALCLP 180

************************************************************

CCR9 ELLYSQVKEEHGIAICTMVYSSDDSTKLKSAVLTLKVILGFFLPFVVMACCYTIIIHTLI 240

NP_001091537 ELLYSQVKEEHGIAICTMVYSSDDSTKLKSAVLTLKVILGFFLPFVVMACCYTIIIHTLI 240

************************************************************

CCR9 QAKKSSKHKALKVTITVLTVFVLSQFPHNCVLLVQTIDAYAMFISSCALSIKIDICFQVT 300

NP_001091537 QAKKSSKHKALKVTITVLTVFVLSQFPHNCVLLVQTIDAYAMFISSCALSIKIDICFQVT 300

************************************************************

CCR9 QTVAFFHSCLNPVLYVFVGERFRRDLVKTLKNLGCISQAQWVSFTRREGSLKLSSMLLET 360

NP_001091537 QTVAFFHSCLNPVLYVFVGERFRRDLVKTLKNLGCISQAQWVSFTRREGSLKLSSMLLET 360

************************************************************

CCR9 TSGALFF 367

NP_001091537 TSGALSF 367

***** *

Amino acid alignment of sequenced CCR9 with published CCR9 sequence (NP_001091537).

Boxed areas indicate transmembrane domains as predicted by SMART analysis [1, 2].

**1. Letunic I, Copley RR, Pils B, Pinkert S, Schultz J, Bork P: SMART 5: domains in the context of genomes and networks. *Nucleic Acids Res* 2006, 34:D257-260.**

**2. Schultz J, Milpetz F, Bork P, Ponting CP: SMART, a simple modular architecture research tool: identification of signaling domains. *Proc Natl Acad Sci U S A* 1998, 95:5857-5864.**
